# The Significance of the WHO/ISH Absolute Cardiovascular Risk Prediction Scores among Recent Stroke Survivors in Ghana—Insights from the PINGS2 multicenter study

**DOI:** 10.21203/rs.3.rs-6175913/v1

**Published:** 2025-03-12

**Authors:** Ansumana S. Bockarie, Nana Kwame Ayisi-Boateng, Samuel Blay Nguah, Lambert Tetteh Appiah, Timothy Fiattor, Sanaa Afriyie-Ansah, Evans MacCready, Victoria Aba Sam, Nathaniel Adusei Mensah, Raelle Tagge, Kwadwo Gyebi Agyenim-Boateng, Micheal Ampofo, Ruth Laryea, Rexford Adu Gyamfi, JH Amuasi, Agnes Amankwaah Arthur, Christiana Duah, Priscilla Abrafi Opare-Addo, Bruce Ovbiagele, Fred Stephen Sarfo, Albert Akpalu

**Affiliations:** University of Cape Coast; University of Science & Technology Hospital; University of Science & Technology Hospital; University of Science & Technology Hospital; Manhyia District Hospital; Cape Coast Teaching Hospital; Cape Coast Teaching Hospital; Cape Coast Teaching Hospital; Komfo Anokye Teaching Hospital; Northern California Institute of Research and Education, USA; Kwadaso SDA Hospital; Komfo Anokye Teaching Hospital; University of Ghana Medical School; Agogo Presbyterian Hospital; Kumasi Centre for Collaborative Research; Ankaase Methodist Hospital; Kumasi South Hospital; Komfo Anokye Teaching Hospital; Northern California Institute of Research and Education, USA; University of Science & Technology; University of Ghana Medical School

**Keywords:** Stroke, Cardiovascular disease, Absolute Risk Score

## Abstract

**Background:**

The leading cause of stroke remains atherosclerotic cardiovascular disease. The World Health Organization/International Society of Hypertension risk score represents an effort to produce a risk assessment tool for atherosclerotic cardiovascular disease that is regionally specific. No previous work has described absolute cardiovascular risk scores among recent stroke survivors in West Africa via this tool.

**Methods:**

A cross-sectional analysis of baseline data from the multicenter, phase III randomized, open-label, clinical trial, Phone-based Intervention under Nurse Guidance II (PINGS-2), was performed. Data from 414 participants who had recently survived a stroke and met the age range compatible with the risk estimation tool were analyzed. The WHO/ISH score was calculated for each participant and categorized into low, moderate, and high/very high CVD risk scores. Demographic data, medical histories, anthropometry, vascular risk profiles, stroke types and severity indices were compared across CVD risk categories. Multivariate logistic regression was performed to further examine variables significantly associated with WHO/ISH CVD risk via univariate analysis.

**Results:**

The mean age of the study population was 58 years (SD = 12), with the majority being male (56.5%). Ischemic strokes (n = 263, 74.3%) were more common than hemorrhagic strokes (n = 78, 22%). Over two-thirds (76.3%) of the participants were estimated to have a low (< 10%) risk of cardiovascular disease in the next 10 years, 14.5% were estimated to have a moderate risk, and only 9.2% were stratified as high or very high risk. The absolute CVD risk score was significantly associated with age, higher income, tobacco use, systolic blood pressure and HBA1c. There was no significant difference in absolute cardiovascular risk by stroke type.

**Conclusion:**

A comparatively lower proportion of Ghanaian stroke survivors were classified as high risk by the WHO/ISH risk score. This raises the question of its appropriateness as a cardiovascular risk assessment tool to drive secondary prevention among this patient population.

**Trial registration:**

NCT04404166. Registered on May 27,2020 at ClinicalTrials.gov

## Background

Cerebrovascular disease is the second most common cause of death in the world and a significant cause of disability (1). The most common cause of the majority of strokes is atherosclerotic cardiovascular disease caused by risk factors such as hypertension, obesity, diabetes and smoking (2). The importance and prevalence of cardiovascular disease (CVD) has driven the need for easily derived predictive and prognostic tools to stratify patients according to their risk over specified time periods.

The Framingham-based risk prediction equations, such as the Framingham coronary risk score (FCRS), probably represent the best-known examples of these tools. Studies have validated these equations in North American and European populations as well as other populations (3–5), including Ghanaians (6). CVD risk assessment tools vary in their performance between various populations and subpopulations (7–11). These inadequacies and the need for population-specific assessment scores have prompted the development of other estimation tools (12–14)

On the basis of the Collaborative Risk Assessment Study (15), the World Health Organization/International Society of Hypertension (WHO/ISH) risk score represents an effort to produce a risk assessment tool that is appropriate for regions such as West Africa, which has hitherto relied on risk prediction tools largely developed in other regions. Risk stratification algorithms for the tool were derived from the mean of risk factors and the average ten-year event rates from countries of each WHO subregion (16). The tool utilizes age, sex, systolic blood pressure, smoking history, the presence or absence of diabetes and total cholesterol to categorize patients aged > 40 years to < 75 years into low, moderate, high, and very high risk of CVD in ten years (17). This broad attempt to create region-specific tools is what some suggest makes the WHO/ISH score most suitable for West African countries such as Ghana (18) and is the CVD risk tool recommended by the Ghana National Cardiovascular Disease Guidelines (19).

There exist algorithms that stratify individuals specifically for risk of stroke, such as the modified Framingham Stroke Risk Score (MFSRS) (20) and the Stroke Investigative Research and Educational Network (SIREN), which were found to have better prediction ability for Africans (21, 22). However, the more common, absolute or total cardiovascular risk predictive models (such as the FCRS and WHO/ISH tools) are still more widely known and more frequently utilized by general clinicians in assessing patients (23). Their greater use in general practice makes an understanding of their relevance and suitability vital. Absolute cardiovascular risk in patients with stroke has also been shown to be of prognostic significance (24–27).

While some work has focused on stratification for the risk of stroke (21, 22), there is a dearth of experience with absolute or total cardiovascular risk stratification among stroke patients in sub-Saharan Africa. There are no studies available from Ghana that specifically assess the relationships between absolute CVD risk scores and population characteristics among stroke survivors or how risk categories are associated with stroke type and severity. This study assessed the relationships between absolute CVD risk and various sociodemographic, clinical and laboratory parameters among patients who have recently suffered a stroke and identified the major contributors to ongoing high CVD risk in this population.

## Methods

### Study Design and Population

This is a cross-sectional analysis of baseline data collected from a recently completed multicenter, phase III randomized, open-label, blinded endpoint clinical trial titled the Phone-based Intervention under Nurse Guidance II (PINGS-2) study. The research protocol (including details of recruitment and sample size estimations) is published elsewhere (28). Eligible participants over the age of 18 who were hypertensive and had survived a stroke less than a month prior were recruited. Study participants were enrolled from 10 hospitals, including four primary-level, three secondary-level and three tertiary-level health facilities, in Ghana across three regions in the middle and lower belts. Baseline demographic data, lifestyle information, medical histories, anthropometric measurements, laboratory investigations and imaging data were obtained. Among the 500 participants recruited for the PINGS-2 study, a subpopulation of 414 participants were within the 40–80 years age range compatible with CVD risk estimation via the WHO/ISH risk calculator. The baseline characteristics of these recent stroke survivors were considered in the present analysis.

### Definitions

The 10-year absolute CVD risk was defined as the probability of an individual experiencing a CVD event over a period of 10 years (16).

Stroke status and type were verified by imaging (computed tomography or magnetic resonance imaging). In instances where imaging data were not available, a locally validated 8-item questionnaire for verifying stroke-free status was used to determine stroke status (29).

Hypertension was defined as having a blood pressure of ≥ 140/90 mmHg or on antihypertensive medications at the time of recruitment (30).

Diabetes mellitus was defined as a medical history of diabetes, treatment for diabetes, a fasting blood glucose level of > 7 mmol/L or a glycated hemoglobin level (HBA1c) > 6.5% (31).

Hyperlipidemia was defined as a self-reported medical history of hyperlipidemia, treatment for hyperlipidemia or laboratory evidence of lipoprotein disorders (32)

Tobacco use was defined as self-reported current or previous use of tobacco products in the preceding year (16).

A reduced estimated glomerular filtration ratio (eGFR) was defined as ≤ 89 ml/min per 1.73 m^2^ (33).

The National Institutes of Health Stroke Scale (NIHSS), a widely validated 11-item clinical assessment tool for the extent of neurological impairment following stroke, (34)was assessed at baseline.

Barthel’s index for functional status, a 10-item scale that measures independence or dependence in the performance of activities of daily living, was assessed at baseline in line with current stroke trial recommendations (35).

The modified Rankin scale, a 7-tier ordinal scale, is a clinical assessment of global disability that serves as a primary outcome measure in most stroke trials (36) and was assessed at baseline in the study population.

### WHO/ISH Risk Scores

WHO/ISH risk scores were derived from the dataset via a validated R plugin (Collins et al., 2017). As in other studies (38–40), the WHO/ISH risk categories were grouped into three categories: low (< 10%), moderate (10% - <20%) and high/very high (> 20%), risk of CVD in ten years. This allows for easier comparison of results with most other absolute CVD scoring systems that use these three more conventional groupings, combining all those with an over 20% risk of CVD in ten years into a single high-risk category (41).

### Statistical analysis

Study data were initially collected via a structured case record form and managed via Research Electronic Data Capture (REDCap) electronic data capture tools hosted at the University of California, USA. The data were subsequently exported to R statistical software (version R 4.4.2; 2024). for analyses. Frequencies and percentages were derived. Means with standard deviations were calculated for those variables conforming with a normal distribution. Univariate ordinal logistic regression was used to describe the relationships between selected variables and cardiovascular risk categories. The Cochran–Armitage test (chi-square trend test) was used to establish evidence of ordered trends or patterns between some categorical variables, whereas the Kruskal–Wallis rank sum test was used to compare the medians of variables when the normal distribution was violated or when variances differed significantly. Significant covariates were then used for multivariate logistic regression. The alpha level of significance was set at 0.05.

### Ethical considerations

Ethical approval for the study was obtained from the Institutional Review Board of the Kwame Nkrumah University of Science and Technology (IRB CHRPE/AP/104/21), as well as from the hospital Ethics and Research Committees of the various study sites. Written informed consent was obtained from all participants and documented. A Data and Safety Monitoring Board (DSMB) monitored study subject safety and ethics (28).

## Results

### Demographic and Clinical Characteristics of the Study Participants by sex:

The stroke survivors included in this subanalysis had a mean age of 58 years (SD=12), with the majority of the 414 participants being male (56.3%). The vast majority were educated, with almost half (48.6%) having obtained secondary education or higher. They were mostly urban dwellers (61.1%), married (68.4%), and nontobacco users (90.6%) (Table 1). The sample had a mean body mass index (BMI) of 26.5 (SD=5.6). At least one-third (33.3%) had diabetes, and a similar proportion (34.3%) had hyperlipidemia. The frequency of a reduced estimated glomerular filtration rate (eGFR) of < 60 ml/min among the participants was 22.6%. There were significant differences in educational status, income, diabetes mellitus status, BMI, tobacco use and waist circumference between the sexes. Imaging-verified ischemic strokes (n=268, 73.4%) were more common than hemorrhagic strokes (n=84, 23%). More than three quarters (76.3%) of the participants were estimated to have a low (<10%) risk of CVD in the next 10 years, 14.5% were estimated to have a moderate risk, and only 9.2% were stratified as high or very high risk. (Table 1).

### Demographic and Clinical Characteristics of the Study Participants by CVD risk category:

Participants’ age increased significantly with increasing CVD risk. Those with a low monthly income and domiciles in rural settings were significantly more likely to have a high CVD risk score. (Table 2). However, sex, educational attainment, and marital status did not vary significantly across the CVD risk categories. Among the cardiovascular risk factors studied, a history of diabetes mellitus, mean systolic blood pressure, mean diastolic blood pressure and tobacco use were significantly associated with high CVD risk. Dyslipidemia and lipid subfractions and total cholesterol, LDL, HDL, and TG levels did not vary significantly along the CVD risk continuum. Notably, the duration of exercise during leisure was significantly longer among those with high CVD risk scores. The stroke type, severity and disability indices were not associated with CVD risk categories. (Table 2).

### Predictors of CVD risk categories among stroke survivors:

Multivariate ordinal logistic regression analysis to further examine variables significantly associated with WHO/ISH CVD risk indicated that age, systolic blood pressure, history of tobacco use and HBA1c remained significant predictors of high WHO/ISH CVD risk (Table 3). Additionally, compared with the lowest income bracket, those earning 251--500 Ghana cedis and >3,000 Ghana cedis monthly had higher CVD risk scores, whereas the location of residence and hours of active leisure were attenuated into nonsignificance upon adjustment for confounders (Table 3).

## Discussion

Using a CVD risk score assessment instrument widely deployed in low-income regions of the globe among a sample of recent Ghanaian stroke survivors, we found that less than 25% had moderate to high CVD risk scores. This study population was recruited from hospitals that serve rural, peri-urban, and urban communities across Ghana. The major implication of our finding is that for a substantial proportion of our population who have experienced a catastrophic clinical event such as stroke, preventative interventions might have not been prioritized on the basis of current guidelines for CVD risk categorization. As expected, we found that age, systolic blood pressure, history of tobacco use, and glycated hemoglobin concentration were the most resilient predictors of high absolute CVD risk among stroke survivors.

The outcomes of categorizing stroke patients using absolute CVD risk scores have yielded a mixed picture across various studies. In a large secondary analysis of data from U.S. veterans (4,391 patients with ischemic stroke), 62.8% (n = 2,759) had high CVD risk (FCRS ≥20%) (42), which was much greater than the 9.2% reported in this study. In another largely North American study among ischemic stroke survivors using the FCRS, the prevalence of high risk was 37% (n=933) (43), which was even higher (84%) than that reported in an Albanian study (44). Although those studies used the FCRS, both the score and the WHO/ISH risk score used in this study are very similar in their use of sex, total blood pressure, systolic blood pressure, diabetes, blood cholesterol level and smoking status in arriving at a composite risk score. The wide difference between the findings of this study and the others may be partly due to the difference in population characteristics between the studies, with this study having a wholly black African population with both ischemic and hemorrhagic strokes. However, even among the ischemic stroke survivors in our sample, only 9.7% had high CVD risk scores. Additionally, the weighting of smoking or tobacco use in the design of the risk equations may lead to under estimation of risk in populations such as the one in our study where smoking is rare. This assertion is partly supported by findings in another West African community where the modified Framingham stroke risk score was found to underestimate stroke risk (11,45).

This study of a relatively younger, predominantly male, married, largely urban dwelling group of mixed ischemic and hemorrhagic stroke survivors revealed associations between CVD risk and several traditional lifestyle, anthropometric, and metabolic risk factors, such as tobacco use, leisure activity, the waist–hip ratio, diabetes mellitus, and HBA1c, via unadjusted analysis. However, no significant associations were found between CVD risk and other traditional risk factors, such as gender, work activity, waist circumference, medical history of dyslipidemia or lipid chemistries. Similarly, Towfighi et al. (2012) reported associations between risk scores and age, diabetes status, smoking status and blood pressure. Additionally, in accordance with this study, Lalo et al. (2023) reported no associations between risk category and education or urban or rural residence. In contrast with this study, however, the former study also revealed an association with cholesterol levels. The relationship between lipids and stroke is complex, and the lack of an association found in this study may be the result of the inclusion of hemorrhagic strokes and several ischemic stroke subtypes that have been found to sometimes not be associated with dyslipidemia (46).

This study’s finding that absolute CVD risk is not associated with the NIHSS score among survivors is similar to that reported by Ovbiagele et al. (2011) among their discharge cohort. Moreover, there was no association between CVD risk and other measures of stroke severity, such as the modified Rankin score and Barthel index. The associations between different risk scores and stroke severity vary, as Park et al. (2015) reported when comparing the FCRS and American College of Cardiology/American Heart Association (ACC/AHA) pooled cohort risk. In that multiethnic cohort, an association was found between the NIH stroke scale score and the former but not the latter. The lack of a significant difference in CVD risk between ischemic, hemorrhagic, and other stroke types found in this study may be partially explained by the fact that these stroke types share many of the same risk factors (47).

### Implications:

First, for stroke survivors in Ghana and other sub-Saharan populations, the WHO/ISH CVD risk calculator may not sufficiently categorize patients into those with high atherosclerotic risk for whom prescriptions of statins and antiplatelets for secondary prevention are indicated. Second, the high percentage of stroke survivors with low CVD risk scores (76%) indicates that using risk scores for primary prevention of CVDs in resource-limited settings might be problematic in identifying those who need lifestyle and drug therapies to reduce their risk. In this young population, a significant proportion may develop a stroke due to a solitary yet dominant risk factor, such as hypertension, which may be undiagnosed, untreated, or uncontrolled. The current risk calculators available may indeed classify these individuals as having low CVD risk and yet come down with a devastating stroke. A rethinking of CVD risk prevention via population-level interventions such as mass polypill use, education on salt reduction, smoking cessation and physical activities rather than a targeted approach using CVD ‘riskometers’ is needed in low- or middle-income countries (LMICs) to curb the epidemic of stroke in these regions.

### Strengths & Limitations

Stroke survivors were recruited from 10 hospitals at various levels of care provision (primary, secondary, and tertiary levels) in the middle and lower belts of Ghana, increasing the generalizability of our study findings. Previous stroke studies in Africa have recruited patients from tertiary medical centers with neuroimaging facilities. In the present study, 3 participants (<1%) who did not have neuroimaging-confirmed stroke were included. Another limitation is the cross-sectional design of the study, which precluded causal associations from being drawn.

## Conclusion

The comparatively lower proportion of Ghanaian stroke survivors classified as high risk by the WHO/ISH risk score, a tool with specific modeling for the West African population, highlights its suitability as a prognostic tool among this stroke survivor cohort. The lack of a significant difference in risk score between different stroke types raises further questions about the interaction between risk factors and etiologies of stroke within the Ghanaian population and indeed other indigenous African stroke populations.

## Data Availability

The data that support the findings of this study are stored on Research Electronic Data Capture (REDCap) electronic data capture tools hosted at the University of California, USA, and are available through the corresponding author upon reasonable request.

## Abbreviations

CVD: Cardiovascular Disease
FCRS: Framingham Coronary Risk Score
WHO/ISH: World Health Organization/International Society of Hypertension
eGFR: estimated glomerular filtration ratio
NIHSS: National Institutes of Health Stroke Scale
BMI: Body mass index
TG: triglyceride
LDL: Low-density lipoprotein cholesterol
HDL: high-density lipoprotein cholesterol
ACC/AHA: American College of Cardiology/American Heart Association
LMIC: Low- or middle-income country
